# A Single Enhancer Regulating the Differential Expression of Duplicated Red-Sensitive Opsin Genes in Zebrafish

**DOI:** 10.1371/journal.pgen.1001245

**Published:** 2010-12-16

**Authors:** Taro Tsujimura, Tomohiro Hosoya, Shoji Kawamura

**Affiliations:** Department of Integrated Biosciences, Graduate School of Frontier Sciences, The University of Tokyo, Kashiwa, Japan; Johns Hopkins, United States of America

## Abstract

A fundamental step in the evolution of the visual system is the gene duplication of visual opsins and differentiation between the duplicates in absorption spectra and expression pattern in the retina. However, our understanding of the mechanism of expression differentiation is far behind that of spectral tuning of opsins. Zebrafish (*Danio rerio*) have two red-sensitive cone opsin genes, *LWS-1* and *LWS-2*. These genes are arrayed in a tail-to-head manner, in this order, and are both expressed in the long member of double cones (LDCs) in the retina. Expression of the longer-wave sensitive *LWS-1* occurs later in development and is thus confined to the peripheral, especially ventral-nasal region of the adult retina, whereas expression of *LWS-2* occurs earlier and is confined to the central region of the adult retina, shifted slightly to the dorsal-temporal region. In this study, we employed a transgenic reporter assay using fluorescent proteins and P1-artificial chromosome (PAC) clones encompassing the two genes and identified a 0.6-kb “LWS-activating region” (LAR) upstream of *LWS-1*, which regulates expression of both genes. Under the 2.6-kb flanking upstream region containing the LAR, the expression pattern of *LWS-1* was recapitulated by the fluorescent reporter. On the other hand, when LAR was directly conjugated to the *LWS-2* upstream region, the reporter was expressed in the LDCs but also across the entire outer nuclear layer. Deletion of LAR from the PAC clones drastically lowered the reporter expression of the two genes. These results suggest that LAR regulates both *LWS-1* and *LWS-2* by enhancing their expression and that interaction of LAR with the promoters is competitive between the two genes in a developmentally restricted manner. Sharing a regulatory region between duplicated genes could be a general way to facilitate the expression differentiation in duplicated visual opsins.

## Author Summary

Among vertebrates, fish may have the most advanced color vision. They have greatly varied repertoires of color sensors called visual opsins, possibly reflecting evolutionary adaptation to their diverse photic environments in water, and are an excellent model to study the evolution of vertebrate color vision. This is achieved by multiplying opsin genes and differentiating their absorption light spectra and expression patterns. However, little is understood regarding how the opsin genes are regulated to achieve the differential expression pattern. In this study, we focused on the duplicated red-sensitive opsin genes of zebrafish to tackle this problem. We discovered an “enhancer” region near the two red opsin genes that plays a crucial role in their differential expression pattern. Our results suggest that the two red opsin genes interact with the enhancer competitively in a developmentally restricted manner. Sharing a regulatory region could be a general way to facilitate the expression differentiation in duplicated visual opsin genes.

## Introduction

Gene duplication is a fundamental step in evolution [1]. Most often, one of the resulting daughter genes simply becomes a pseudogene and may be eventually lost from the genome due to functional redundancy between the duplicates and reduction of selective constraint to maintain its function. However, observation of another fate for duplicated genes, such as acquisition of a new function (neofunctionalization) or subdivision of parental gene function between daughter genes (subfunctionalization), implies an evolutionary advantage by the process [2]. Subfunctionalization often involves differentiation of expression pattern between daughter genes and has been a subject of intense scrutiny to understand the regulatory mechanism to achieve the differentiation [3]–[5].

In vertebrates, color vision is enabled by the presence of multiple classes of cone visual cells in the retina, each of which has a different absorption spectrum. The absorption spectrum of a visual cell is mainly determined by the visual pigment it contains. A visual pigment consists of a protein moiety, visual opsin, and a photo-sensing chromophore, either 11-*cis* retinal or 11-*cis* 3,4-dehydroretinal [6]. The five types of visual opsins found among extant vertebrates are RH1 (rod opsin or rhodopsin) and four types of cone opsins: RH2 (RH1-like, or green), SWS1 (short wavelength-sensitive type 1, or ultraviolet-blue), SWS2 (short wavelength-sensitive type 2, or blue) and M/LWS (middle to long wavelength-sensitive, or red-green) [7]. The SWS2 and M/LWS type genes are closely located on the same chromosome [8]–[10] and could represent the most ancient gene duplication in vertebrate visual opsin genes, from which other types could have arisen through whole-genome duplications and subsequent gene losses in early vertebrate evolution [7], [11]–[13]. Thus, visual opsin genes represent an excellent case of gene duplication to study the mechanism of neofunctionalization (in absorption spectrum) and subfunctionalization (in expression pattern). While the spectral tuning mechanism of visual opsins has been intensively studied [14]–[18], the regulatory mechanism of their expression differentiation, especially that of cone opsins, has been less explored.

Among vertebrates, fish are known to possess a rich and varied repertoire of visual opsins, including two or more opsin subtypes within the five types by further gene duplications [19]–[21], presumably reflecting their evolutionary adaptation to diverse aquatic light environments [22]. In fish, the eyes continue to grow throughout their lifetime by adding new cells to the peripheral zones, such that the peripheral cells are developmentally younger than central cells [23], [24]. Thus, in the fish retina the timing of gene expression is partly reflected in the region of expression in the retina. All visual opsin genes have been isolated and characterized for zebrafish (*Danio rerio*) [25], medaka (*Oryzias latipes*) [26] and cichlids (Family Cichlidae) [27]–[30]. Among them, the expression pattern of visual opsin genes has been best documented for zebrafish.

Zebrafish have nine visual opsin genes consisting of two M/LWS (red), four RH2 (green), and single-copy SWS1 (UV), SWS2 (blue) and RH1 (rod) opsin genes [25]. The red, green, UV and blue opsin genes are expressed in the long-member of double cones (LDCs), the short-member of double cones (SDCs), the short single cones (SSCs) and the long single cones (LSCs), respectively, which are arranged in a regular mosaic pattern in the retina [31], [32]. The two red opsin genes, *LWS-1* and *LWS-2*, are arrayed in a tail to head manner, in this order, and encode photopigments with wavelengths of maximal absorption (λmax) at 558 and 548 nm, respectively [25]. The four green opsin genes, *RH2-1*, *RH2-2*, *RH2-3* and *RH2-4*, are also arrayed in a tail to head manner, in this order, and encode photopigments with λmax at 467, 476, 488, and 505 nm, respectively [25]. In both red and green opsins, expression of longer-wave subtypes occurs later in development and is confined to the peripheral, especially ventral-nasal region of the adult retina, whereas expression of shorter-wave subtypes occurs earlier and is confined to the central region of the adult retina, shifted slightly to the dorsal-temporal region [33]. It remains largely unknown how subtypes of an opsin class are directed to express in different regions of the retina while keeping the cell type identical between them. Thus, the zebrafish visual opsins are an excellent model to study the regulatory mechanism of not only cell-type specific expression of opsin types, but also developmental-stage (and thus retinal-region) specific expression of opsin subtypes.

With the feasibility to employ transgenic technology, *cis*-regulatory regions relevant to the cell-type specific expression of opsin types have been elucidated using a living color reporter such as the green fluorescent protein (GFP) for zebrafish single-copy opsin genes (*i.e.*, rod opsin [34]–[37], UV opsin [38], [39] and blue opsin genes [40]). A regulatory region relevant to not only cell-type specific but also retinal-region specific expression of opsin subtypes has also been reported for the zebrafish green opsin genes [41]. In the present study, we focus on the zebrafish red opsin genes, *LWS-1* and *LWS-2*, and report a *cis*-regulatory region, “LWS-activating region” (LAR), which is relevant to their expression differentiation.

## Results

### P1 artificial chromosome (PAC) clones contain a sufficient regulator for expression of the two red opsin genes of zebrafish

**Figure 1:**
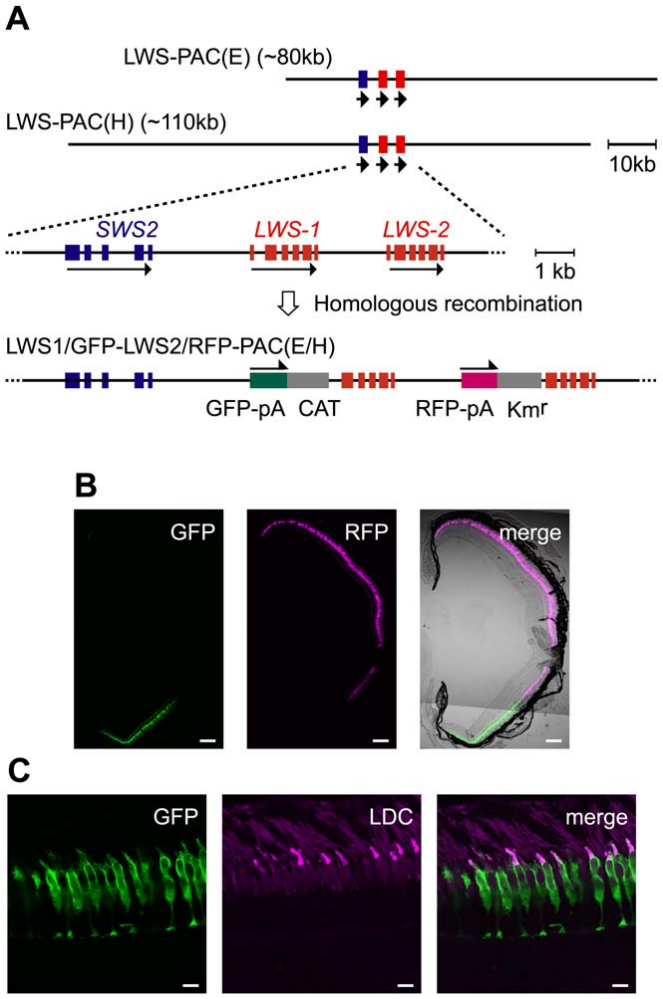
Recapitulation of the *LWS-1* and *LWS-2* expression in the zebrafish retina by the fluorescent markers in the PAC clones. (A) Construction of the LWS1/GFP-LWS2/RFP-PAC clones. The two PAC clones, LWS-PAC(E) and LWS-PAC(H), both encompass the two red opsin genes, *LWS-1* and *LWS-2*, in addition to the blue opsin gene, *SWS2*. In the expanded view, the blue and red boxes indicate the exons of the blue and red opsin genes, respectively. The orientation of transcription is given by the arrows for each gene. In both the LWS1/GFP-LWS2/RFP-PAC(E) and LWS1/GFP-LWS2/RFP-PAC(H) clones, the first exons of *LWS-1* and *LWS-2* were replaced with the GFP-polyA-CAT and RFP-polyA-Km^r^ cassettes by site-specific homologous recombination, respectively. (B) A transverse section of the retina of an adult Tg(LWS1/GFP-LWS2/RFP-PAC(E))#1229 fish. The dorsal side is oriented at the top of each panel and the ventral side is at the bottom. GFP (green) is expressed in the ventral region (left), whereas RFP (magenta) is expressed in the central to dorsal region (middle). The right panel shows the merge of the left two panels with the transmitted light image taken by the differential interference contrast microscopy (DIC image). (C) A vertical section of the photoreceptor layer in the ventral retina of an adult Tg(LWS1/GFP-LWS2/RFP-PAC(H))#430 fish. The outer segments of LDCs are immunostained with an antibody against the zebrafish red opsin (magenta). Note that the GFP (green) is found mostly in the cell body of LDCs. Scale bars  = 100 µm for (B), 10 µm for (C).

In the two PAC clones we obtained [LWS-PAC(E) and LWS-PAC(H)], the first exons of *LWS-1* and *LWS-2* were replaced after their initiation codons with DNA segments encoding green and red fluorescent proteins (GFP and RFP), respectively (Figure 1A). The modified clones were designated LWS1/GFP-LWS2/RFP-PAC(E) and LWS1/GFP-LWS2/RFP-PAC(H), respectively. One transgenic zebrafish line was established using each construct: Tg(LWS1/GFP-LWS2/RFP-PAC(E))#1229 and Tg(LWS1/GFP-LWS2/RFP-PAC(H))#430.

Adult fish of both lines expressed RFP in the central-dorsal-temporal region of the retina and GFP in the peripheral-ventral-nasal region of the retina circumscribing the RFP region (Figure 1B). It was also confirmed that the expression was specific to the LDCs, which were immunostained by an antibody against the zebrafish red opsin (Figure 1C). Thus, in both transgenic lines, the expression of GFP and RFP reporter genes recapitulated the expression of *LWS-1* and *LWS-2*, respectively, demonstrating that both the LWS-PAC(E) and LWS-PAC(H) clones contain sufficient regulatory region(s) for the proper expression of the two red opsin genes.

### The upstream region of *LWS-1* regulates expression of not only *LWS-1*, but also *LWS-2*

**Figure 2:**
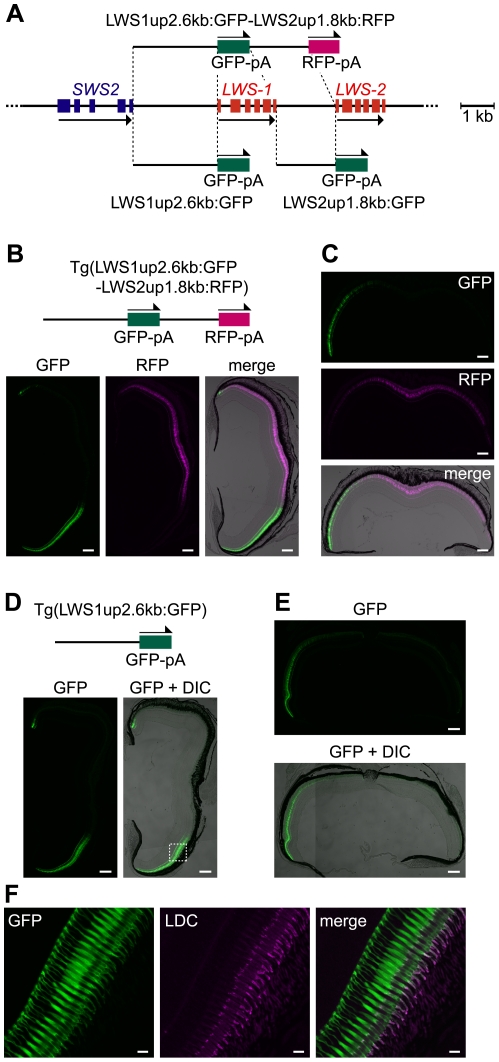
Regulatory ability of the 5′ flanking regions of *LWS-1* and *LWS-2*. (A) Schematic representation of the GFP or RFP expression constructs. (B) A transverse section of an adult Tg(LWS1up2.6kb:GFP-LWS2up1.8kb:RFP)#1631 retina. The dorsal side is oriented at the top of each panel and the ventral side is at the bottom. The left and middle panels show the GFP (green) and RFP (magenta) images, respectively. The right is the merge of the two panels with the DIC image. (C) A horizontal section of the retina from the same adult individual shown in (B). The nasal side is shown on the left and the temporal side is on the right. The upper and middle panels show the GFP (green) and RFP (magenta) images, respectively, and the lower one is the merge of the two panels with the DIC image. (D, E) Transverse (D) and horizontal (E) sections of an adult Tg(LWS1up2.6kb:GFP)#1509 retina. The left (D) and top (E) panels show images of GFP signals (green) and the right (D) and bottom (E) panels show their overlays with the DIC images. The dorsal side is oriented at the top of each panel and the ventral side is at the bottom in (D). The nasal side is oriented on the left and the temporal side is on the right in (E). (F) A vertical view of the photoreceptor layer magnified from the dashed rectangle in (D). The left panel shows the GFP signals (green) and the middle panel shows the signals of immunostaining against the zebrafish red opsin in the outer segments of LDCs (magenta). The right panel shows a merge of the left two panels. The overlap of the two signals appears as white. Scale bars  = 100 µm for (B–E), 10 µm for (F).

Next, we used only the intergenic region between the stop codon of *SWS2* and the initiation codon of *LWS-1*, designated LWS1up2.6kb, and the region between the stop codon of *LWS-1* and the initiation codon of *LWS-2*, designated LWS2up1.8kb. We created a double-reporter construct consisting of the LWS1up2.6kb, GFP reporter, LWS2up1.8kb and RFP reporter, in this order (LWS1up2.6kb:GFP-LWS2up1.8kb:RFP, Figure 2A), and obtained three transgenic lines: Tg(LWS1up2.6kb:GFP-LWS2up1.8kb:RFP)#1464, 1631, and 1640. In two of the three lines, #1631 (Figure 2B, 2C) and #1640, the GFP and the RFP recapitulated the expression patterns of *LWS-1* and *LWS-2*, respectively. In the third line, #1464, the expression of RFP was weaker and sparser but still confined to the central region of the retina and the expression of GFP appeared to be identical to the other two lines (Figure S1). The expression pattern of *LWS-1* was also recapitulated when only the LWS1up2.6kb was used with GFP (LWS1up2.6kb:GFP) in all three transgenic lines obtained: Tg(LWS1up2.6kb:GFP)#1508, 1509 (Figure 2A, 2D–2F), and 1515. On the other hand, when only the LWS2up1.8kb was used with GFP (LWS2up1.8kb:GFP), no GFP signal was observed in the transgenic line obtained: Tg(LWS2up1.8kb:GFP)#1433. These results suggest that the LWS1up2.6kb contains a regulatory region not only for *LWS-1*, but also for *LWS-2*.

### An “LWS-activating region” (LAR) was found in the upstream region of *LWS-1*

**Figure 3:**
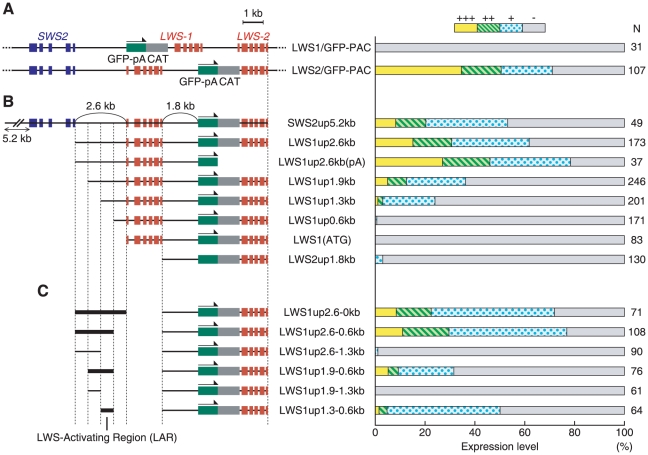
Localization of LAR. The GFP expression constructs used in the transient transgenic assay are depicted with their names on the left side and the corresponding expression levels of GFP in the retina at 7 dpf are indicated in the histogram on the right side. The histogram shows the percentage of eyes graded into four levels (+++, ++, + and −) according to the number of GFP-expressing cells in the retina. The numbers to the right of the histogram show the total number of eyes examined for each of the constructs. (A) The entire PAC clones were tested using LWS1/GFP-PAC and LWS2/GFP-PAC. (B) A series of DNA regions from the LWS2/GFP-PAC were tested. (C) A variety of DNA segments from the LWS1up2.6kb region (segment names given in the panel) were co-injected with the LWS2up1.8kb-GFP-*LWS-2* region.

In order to search for the regulatory region from the LWS1up2.6kb, we employed the transient transgenic assay in which the regulatory activity of a GFP-reporter construct was evaluated in the fish injected with the construct. This was done by examining the incidence of fish bearing GFP-expressing eyes at a larval stage. As in previous studies [39]–[41], the expression level of GFP was graded into four categories, +++, ++, +, −, at 7 days post-fertilization (dpf) (Figure 3). First, we used a whole PAC clone, LWS-PAC(E), and modified it to LWS1/GFP-PAC(E) and LWS2/GFP-PAC(E), in which the first exon of *LWS-1* and *LWS-2*, respectively, was replaced after its initiation codon with GFP-encoding DNA (Figure 3A left). We confirmed that the GFP expression pattern from the two PAC constructs was consistent with the expression patterns of *LWS-1* and *LWS-2*, respectively, at the larval stage (Figure 3A right) (*i.e.*, *LWS-2* is expressed predominantly and *LWS-1* is expressed only faintly in the retina [33]).

Next, as shown in Figure 3B left, we isolated from LWS2/GFP-PAC(E) a series of DNA regions consisting basically of the LWS2up1.8kb-GFP-*LWS-2* region and varying ranges of its upstream region. The GFP signal was apparent when the upstream region contained 1.3-kb or more upstream of *LWS-1*, but was almost undetectable when it contained 0.6-kb or less upstream of *LWS-1* or when only the LWS2up1.8kb-GFP-*LWS-2* region was used (Figure 3B right). This implies that the LWS2up1.8kb region does not contain a sufficient regulatory region for the expression of *LWS-2*, consistent with the absence of a GFP signal in the transgenic line Tg(LWS2up1.8kb:GFP) described above. This also suggests that the regulatory region is located in the 0.6-kb region between 1.3-kb and 0.6-kb upstream of *LWS-1*.

To test if the 0.6-kb region plays a regulatory role by itself for the expression of *LWS-2*, a coinjection protocol was employed using mixed concatamers of separate DNA fragments formed upon integration into the genome [42]. The LWS2up1.8kb-GFP-*LWS-2* region was injected together with a variety of DNA segments from the LWS1up2.6kb region (Figure 3C left). GFP expression was apparent in the retina only when the segment contained the 0.6-kb region (Figure 3C right). We thus designated the 0.6-kb region as an “LWS-activating region” (LAR).

### The LAR enhances LDC–specific gene expression in a position-dependent manner relative to genes

**Figure 4:**
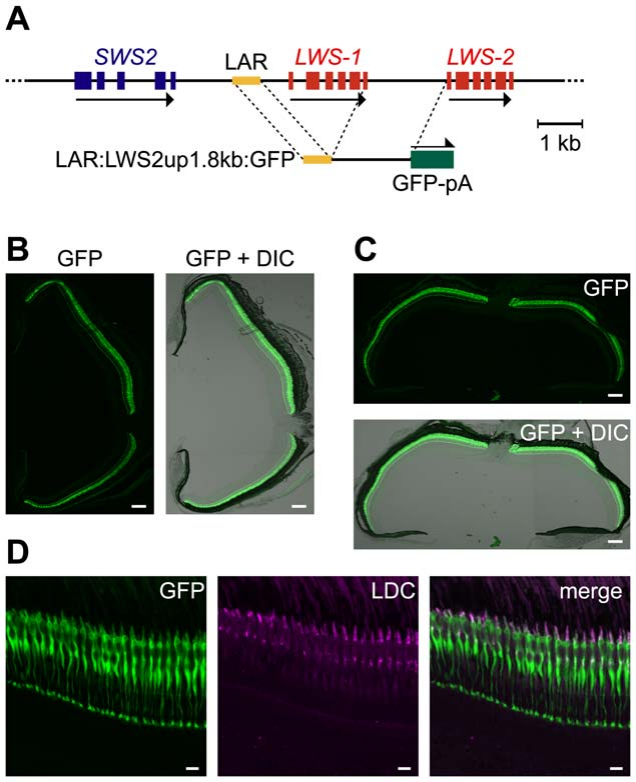
LDC–specific expression of GFP in the entire retina by LAR:LWS2up1.8kb:GFP. (A) Schematic representation of the construction of LAR:LWS2up1.8kb:GFP. (B, C) Transverse (B) and horizontal (C) sections of an adult Tg(LAR:LWS2up1.8kb:GFP)#1499 retina. The left (B) and top (C) panels show images of GFP signals (green) and the right (B) and bottom (C) panels show their overlays with the DIC images. The dorsal side is oriented at the top of each panel and the ventral side is at the bottom in (B). The nasal side is on the left and the temporal side is on the right in (C). (D) A vertical and expanded view of the photoreceptor layer from the same eye sample as shown in (B). The left panel shows the GFP signals (green) and the middle panel shows the signals of immunostaining against the zebrafish red opsin in the outer segments of LDCs (magenta). The right panel shows a merge of the left two panels. The overlap of the two signals appears as white. Scale bars  = 100 µm for (B, C), 10 µm for (D).

Using a DNA construct consisting of LAR and LWS2up1.8kb:GFP, designated LAR:LWS2up1.8kb:GFP (Figure 4A), we obtained five transgenic lines: Tg(LAR:LWS2up1.8kb:GFP)#1481, 1491, 1496, 1499, and 1501. In one line, #1499, a GFP signal was observed specifically in the LDCs but across the entire outer nuclear layer, not confined to the central-temporal-dorsal region (Figure 4B–4D). The absence of retinal region specificity is in sharp contrast to the case in which the double reporter construct, LWS1up2.6kb:GFP-LWS2up1.8kb:RFP, was used (Figure 2B, 2C). This suggests that the relative position of the LAR to the gene is relevant to the regional specificity of the retina.

In another line of Tg(LAR:LWS2up1.8kb:GFP)#1501, the GFP signal also appeared throughout the retina, but was sparser (Figure S2A). At a finer level, the signal appeared not only in LDCs but also weakly in some bipolar cells (Figure S2B). In the other three Tg(LAR:LWS2up1.8kb:GFP) lines, the GFP signal was not detectable. This instability of the reporter signal among the transgenic lines could be attributed not only to the general effect of their insertion sites in the genome, but also to the dependency of LAR to work cooperatively with its adjacent regions in the LWS1up2.6kb and LWS2up1.8kb. Consistently, as in the transient transgenic assay shown in Figure 3B, GFP expression level was much higher when the entire LWS1up2.6kb region was used than when only the proximal 1.3 kb region was used.

To examine if the LDC-specificity of the GFP expression was attributed to LAR itself, we tested the 564-bp adjacent upstream region of a non-retinal keratin 8 gene [43], designated *krt8*up564bp [41]. The *krt8*up564bp induces gene expression specifically in the epithelial tissues, but not in the retina, and has been used for enhancer trapping as a basal promoter [44]. When *krt8*up564bp was conjugated to the LAR and GFP reporter (LAR:*krt8*up564bp:GFP), no GFP expression was observed in the retina of the two transgenic lines obtained: Tg(LAR:*krt8*up564bp:GFP)#1469 and 1477. This is in sharp contrast to the case in which *krt8*up564bp was conjugated to the RH2-LCR and GFP expression was observed in the SDCs throughout the zebrafish retina in our previous study [41]. This suggests that LAR itself is not capable of determining the cell-type specificity of gene expression, unlike RH2-LCR, but works as an enhancer which interacts with cell-type determining regions that should reside in both LWS1up2.6kb and LWS2up1.8kb.

### The LAR affects the expression level of both *LWS-1* and *LWS-2*

**Figure 5:**
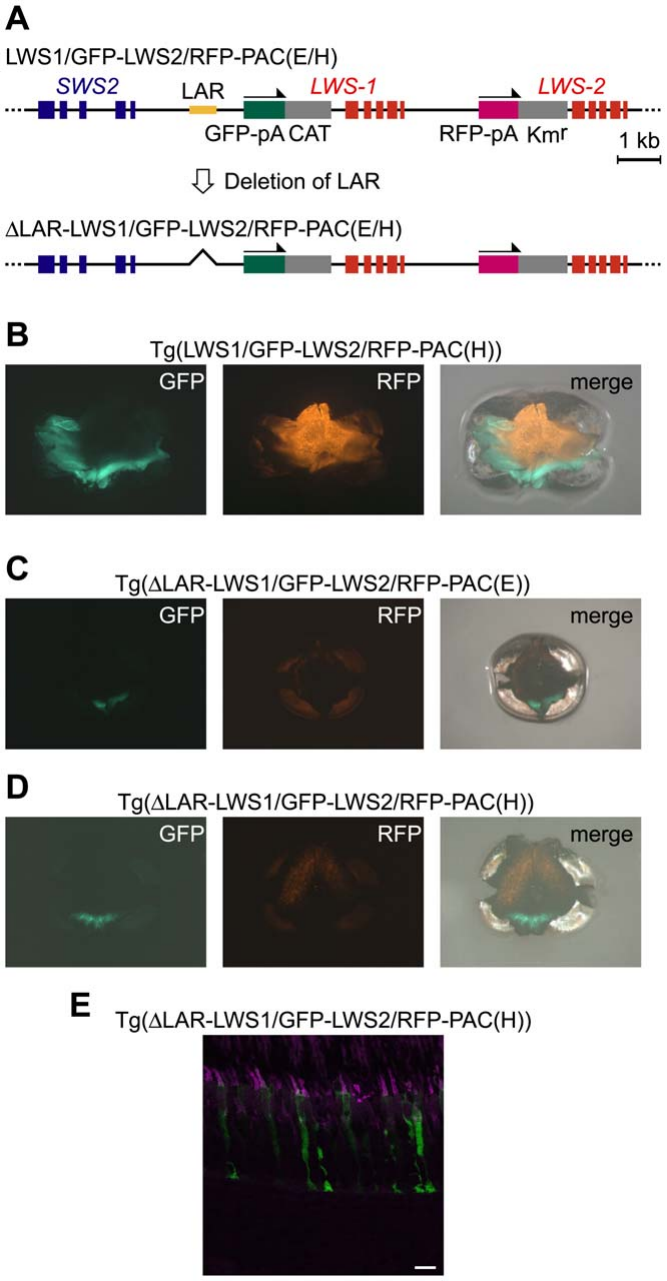
A negative effect of LAR deletion in the reporter gene expression from the LWS1/GFP-LWS2/RFP-PAC clones. (A) Schematic representation of the construction of ΔLAR-LWS1/GFP-LWS2/RFP-PAC(E) and (H). (B, C, D) Images of a whole mount retina of an adult Tg(LWS1/GFP-LWS2/RFP-PAC(H))#430 (B), Tg(ΔLAR-LWS1/GFP-LWS2/RFP-PAC(E))#1143 (C), and Tg(ΔLAR-LWS1/GFP-LWS2/RFP-PAC(H))#1107 (D) fish. The dorsal side is oriented at the top of each panel, and the nasal side is on the left. The GFP signals appear as green and the RFP signals appear as orange. The right panel is the overlay of the left and center panels. (E) A vertical and expanded view of the photoreceptor layer from the same transgenic line as shown in (D). The LDCs were immunostained with the antibody against the zebrafish red opsin (magenta). The GFP signals appear as green. Overlap of the two signals appears as white. Scale bars  = 10 µm.

Next, we removed the LAR from LWS1/GFP-LWS2/RFP-PAC(E) and LWS1/GFP-LWS2/RFP-PAC(H) (designated ΔLAR-LWS1/GFP-LWS2/RFP-PAC(E) and ΔLAR-LWS1/GFP-LWS2/RFP-PAC(H), respectively) (Figure 5A). Two transgenic lines were found for each of the two constructs: Tg(ΔLAR-LWS1/GFP-LWS2/RFP-PAC(E))#1143 (Figure 5C) and 1166 and Tg(ΔLAR-LWS1/GFP-LWS2/RFP-PAC(H))#1107 (Figure 5D, 5E) and 1100. All four of these transgenic lines showed a similar expression pattern of the reporters in the retina with the LAR-bearing Tg(LWS1/GFP-LWS2/RFP-PAC(E)) or Tg(LWS1/GFP-LWS2/RFP-PAC(H)) (Figure 5B) line. The reporter-expressing cells were confined to LDSs (Figure 5E). The GFP and RFP signals were observed in the ventral and dorsal regions of the retina, respectively (Figure 5B–5D). However, the fluorescent signal in each cell was lowered. The number of the reporter-expressing cells decreased and their spatial distribution was restricted to a narrow range in both of these regions (Figure 5B–5E). These results support the deduced role of LAR as an enhancer but not as the cell-type determining factor from the experiments thus far. This experiment also provided the first direct evidence that *LWS-1* expression is affected by LAR.

## Discussion

The present study identified a 0.6-kb regulatory region, named LAR, for the expression of the duplicated red opsin genes of zebrafish, *LWS-1* and *LWS-2*, in the upstream of the gene array. The LAR functions to enhance the LDC-specific expression of both genes but does not determine the cell-type specificity of the gene expression. The regulatory region for the cell-type specificity of the gene expression appears to reside in the 2.6-kb and 1.8-kb upstream regions of the two genes. The relative position of LAR to a gene is relevant to the retinal region specificity of the expression of the gene.

In the primate L/M opsin genes, the locus control region (LCR) is located at ∼3.5-kb upstream of the gene array and is necessary for the expression of both L and M opsin genes [45], [46]. Although there is no clear overall similarity between the zebrafish LAR and primate L/M opsin LCR, LAR contains two OTX (A/GGATTA) and one OTX-like (TGATTA) sequences (Figure S3) which are also present in the primate L/M opsin LCR [47], [48]. These sequences, or their reverse complement sequences, are the binding sites of the cone-rod homeobox (Crx) protein, a member of the Otx family of the paired-like homeodomain proteins and a key *trans*-acting regulatory factor responsible for the gene expression in the retina and pineal organ [47], [48]. The mammalian Crx is produced predominantly in both the retinal photoreceptors and pineal cells and regulates expression of retinal photoreceptor-specific genes and of pineal-specific genes [47]–[50]. In zebrafish, Otx5, a paralog of Crx, is produced in the retina and pineal organ and regulates genes that show circadian expression in the pineal organ [51]. The OTX or OTX-like sequences have also been found in the upstream region of the zebrafish *SWS2* [40] and in the RH2-LCR [41]. Thus, the LAR could be orthologous to the primate L/M opsin LCR and also be paralogous to the *SWS2* regulatory region and the RH2-LCR (see ref. [12] for a similar discussion).

The primate L/M opsin LCR interacts with only the most proximal or the second proximal gene of the array, often L and M opsin genes respectively, through their proximal promoters [46], [52], [53]. The choice of the promoters by the LCR is largely a stochastic process [54], [55]. These characteristics enable the mutually exclusive expression of the L and M opsin genes and nearly a random distribution of the L and M cone photoreceptor cells in the primate retina. In zebrafish, the expression of *LWS-1* and *LWS-2* is also nearly mutually exclusive in the retina [33]. Unlike the primate L/M opsin system, however, the expression of the two zebrafish red opsin genes is temporally and spatially organized and not random in the retina [33]. Whereas expression of *LWS-2* is first observed at 40 hours post-fertilization (hpf) and spread throughout the retina by 72 hpf, initial expression of *LWS-1* is observed at 3.5–5.5 days post-fertilization (dpf) in the marginal side of the ventral retina [33]. In sexually mature adults, *LWS-2* is expressed in the central-dorsal-temporal region of the retina. Expression of *LWS-1* is complementary to the *LWS-2* observed in the peripheral-ventral-nasal region of the rest of the retina, although cells at the boundary of the two fields appear to express both gene subtypes and *LWS-1* is sparsely expressed in the *LWS-2* zone [33].

In this study, the spatially restricted patterns of gene expression were recapitulated by fluorescent reporters for both *LWS-1* and *LWS-2* in the adult retina of Tg(LWS1up2.6kb:GFP-LWS2up1.8kb:RFP) (Figure 2B, 2C). The expression pattern of *LWS-1* was also recapitulated in Tg(LWS1up2.6kb:GFP) (Figure 2D, 2E), whereas that of *LWS-2* was not, and GFP was expressed throughout the adult retina in Tg(LAR:LWS2up1.8kb:GFP) (Figure 4B, 4C). This suggests that the LWS1up2.6kb contains a region susceptive to a developmental control that represses gene expression in the early stage or activates it in the later stage in LDCs, while the LWS2up1.8kb allows LDC-specific expression throughout development with the aid of LAR. This also suggests that LAR, which is shared by *LWS-1* and *LWS-2*, interacts with the *LWS-2* promoter during the time *LWS-1* expression is repressed (or not activated) in the early stage and then interacts with the *LWS-1* promoter once the *LWS-1* expression is enabled. This preference in interaction of LAR for *LWS-1* over *LWS-2* could be attributed to the closer distance of LAR to *LWS-1*, as in the case of the primate L/M opsin LCR [46], [52], [53] and the zebrafish RH2-LCR [41].

Sharing a regulatory region among duplicated genes is a common feature among the zebrafish M/LWS (red) and RH2 (green) and the primate M/LWS (L and M) opsin genes. This system should be advantageous in facilitating differential (*i.e.*, mutually exclusive) expression of duplicated opsin genes by using the regulatory region in a competitive manner between the duplicated genes. If the competition is largely stochastic, an intermingled pattern of photoreceptor cells expressing different daughter genes can be expected in the retina, as in the case of primate L/M opsin genes. The trichromatic color vision is enabled by this stochastic-type system in primates. If the competition is developmentally controlled, for example, so that the regulatory region interacts with a proximal gene in an early stage and shifts the interaction target to a distal gene, the proximal gene would be expressed in the central region and the distal gene in the peripheral region of the retina as in the case of the zebrafish green opsin genes. In the case of the zebrafish red opsin genes, the interaction would start with the distal gene and switch to the proximal gene. In fish, such a control is feasible because the retina continues to grow throughout their lifetime by adding new cells to the peripheral zone [24]. Expression of different opsin genes among different retinal regions results in sights with varying wavelength sensitivity as a function of visual angles, which could be advantageous in the aquatic light environment where wavelength composition differs depending on directions [56]. This could explain why many examples of gene duplication have been found in fish visual opsin genes. Further studies of the regulatory mechanism of differential expression of fish visual opsin genes should contribute to our understanding of the adaptive significance of gene duplications in general.

## Materials and Methods

### Ethics statement

All animal protocols were approved by the University of Tokyo animal care and use committee.

### LWS-PAC clones

Through the screening service of the Resource Center Primary Database (RZPD, Germany; https://www.rzpd.de) of a zebrafish PAC library (no. 706, originally created by C. Amemiya), two clone DNAs (BUSMP706E19271Q9 and BUSMP706H1397Q9), designated LWS-PAC(E) and LWS-PAC(H), were obtained using the *LWS-2* cDNA as a probe. Both clones encompass *SWS2*, *LWS-1* and *LWS-2* in their ∼80-kb and ∼110-kb inserts, respectively (Figure 1A). Sequencing both ends of the inserts revealed that the nucleotide sequences of LWS-PAC(E) and LWS-PAC(H) correspond to the nucleotide positions 25222648–25311454 and 25174505–25295119 of chromosome 11 in the Ensembl zebrafish assembly version 8, respectively (http://www.ensembl.org/Danio_rerio/Info/Index).

### Modification of the LWS-PAC clones by homologous recombination

The I-SceI meganuclease system [57] was used for efficient transgenesis of the PAC-derived constructs. Two I-SceI recognition sites (5′-TAGGGATAACAGGGTAAT-3′) were introduced into the vector backbone of the LWS-PAC clones as follows. The ampicillin-resistance (Amp^r^) gene was PCR-amplified from the pUC18 plasmid using primers harboring the I-SceI recognition site at their 5′ ends to create the I-SceI-Amp^r^-I-SceI segment (see “I-SceI-Amp^r^-I-SceI” in Table S1 for primers). The I-SceI-Amp^r^-I-SceI segment was inserted into the EcoRV site of pBluescript II (SK-) plasmid vector (Stratagene, Tokyo). The I-SceI-Amp^r^-I-SceI segment was isolated from the pBluescript clone using primers harboring the flanking sequences of the kanamycin-resistance (Km^r^) gene site of the LWS-PAC clones to create the I-SceI-Amp^r^-I-SceI cassette (see “Km^r^<>I-SceI-Amp^r^-I-SceI” in Table S1 for primers). The Km^r^ of the LWS-PAC clones was replaced with the I-SceI-Amp^r^-I-SceI cassette by the site-specific homologous recombination system coupled with drug selection using the *E. coli* strain EL250 [58] as in our previous study [41].

The first exon after the initiation codon of *LWS-1* and *LWS-2* in the LWS-PAC clones was replaced with the GFP or the RFP gene as follows. The chloramphenicol acetyl transferase (CAT) and the Km^r^ gene fragments were PCR-amplified from pBR328 and pCYPAC6 plasmids, respectively (see “CAT” and “Km^r^” in Table S1 for primers). The CAT gene was inserted into the pEGFP-1 plasmid vector (BD Biosciences Clontech, Tokyo) at the AflII site, which is located immediately downstream of the SV40 polyadenylation signal (polyA), linked downstream of the GFP coding sequence to create the GFP-polyA-CAT segment. Similarly, the Km^r^ gene was inserted into the pDsRed-1 or pDsRed-Express-1 plasmid vector (BD Biosciences Clontech, Tokyo) at the AflII site to create the RFP-polyA-Km^r^ segment. For the LWS1/GFP-LWS2/RFP-PAC(H), the pDsRed-1 was used. For the other RFP-containing constructs (LWS1/GFP-LWS2/RFP-PAC(E), ΔLAR-LWS1/GFP-LWS2/RFP-PAC(E) and ΔLAR-LWS1/GFP-LWS2/RFP-PAC(H)), the pDsRed-Express-1 was used. The GFP-polyA-CAT segment was isolated from the pEGFP-1 construct by PCR using primers harboring the flanking sequences of the exon 1 of *LWS-1* or *LWS-2* to create the GFP-polyA-CAT cassette (see “LWS-1<>GFP-polyA-CAT” and “LWS-2<>GFP-polyA-CAT” in Table S1 for primers). The RFP-polyA-Km^r^ segment was isolated from the pDsRed-1 or the pDsRed-Express-1 construct by PCR using primers harboring the flanking sequences of the exon 1 of *LWS-2* to create the RFP-polyA-Km^r^ cassette (see “LWS-2<>RFP-polyA-Km^r^” in Table S1 for primers). These cassettes were replaced with the exon 1 of *LWS-1* or *LWS-2* in the LWS-PAC clones by the site-specific homologous recombination system in EL250.

The LAR was removed from the LWS-PAC clones by the site-specific homologous recombination system and by the *flpe*-FRT recombination system for excision of a DNA region sandwiched by FRT sequences in EL250 [41], [58] as follows. The CAT gene was PCR-amplified from the pBR328 using primers harboring the FRT sequences to create the FRT-CAT-FRT segment (see “FRT-CAT-FRT” in Table S1 for primers). The FRT-CAT-FRT segment was inserted into the EcoRV site of pBluescript II (SK-) plasmid. Then, the FRT-CAT-FRT segment was isolated by PCR using primers harboring the flanking sequences of the LAR to create the FRT-CAT-FRT cassette (see “LAR<>FRT-CAT-FRT” in Table S1 for primers). The LAR was replaced with the FRT-CAT-FRT cassette in the LWS-PAC clones by the site-specific homologous recombination system in EL250. The FRT-CAT-FRT cassette was then excised from the modified LWS-PAC clones in EL250 by the *flpe*-FRT recombination system for excision of a DNA region sandwiched by FRT sequences, leaving one FRT sequence in this region of the clones [41], [58].

### Reporter constructs for Tol2-mediated transgenesis

A plasmid construct, pT2AL200R150G [59], was modified as follows. The pT2AL200R150G contains a GFP-expression cassette between XhoI and BglII sites surrounded respectively by the L200 and R150 minimum recognition sequences of the Tol2 transposase. The Tol2 transposase excises the DNA region between the recognition sequences from the plasmid and integrates it into the host genome as a single copy with the recognition sequences attached as in the plasmid [60]. The GFP-expression cassette contains a promoter sequence of a ubiquitously expressed gene (the *Xenopus* elongation factor (EF) 1α), the rabbit β-globin intron, GFP gene and the SV40 polyA signal. The construct contains two Not I sites, one in the junction between the GFP gene and the SV40 polyA and another upstream of the L200 in the vector backbone. We first removed the Not I site in the vector backbone by eliminating a DNA segment between a Sac I site and L200 encompassing the NotI site. Next, we removed the promoter from the construct by replacing the region from the EF1α promoter to the GFP gene (from XhoI to NotI sites) with a DNA segment in the pEGFP-1 vector consisting of a part of the multiple cloning site (MCS) and the GFP gene (from XhoI to NotI sites of pEGFP-1). Finally, we replaced the SV40 polyA signal in the construct (from NotI to BglII sites) with the polyA signal sequence derived from the herpes simplex virus thymidine kinase (HSV-TK), which was PCR-isolated from the pEGFP-1 vector using a forward primer harboring NotI site and a reverse primer harboring BglII site (“HSV-TK-polyA” in Table S2). This modified construct was designated pT2GFP-TKPA. The replacement of the polyA signal from SV40 to HSV-TK was done to facilitate, in a later stage, the insertion of a DNA fragment containing the SV40 polyA in an appropriate orientation into the pT2GFP-TKPA by avoiding a possible interaction between the two SV40 polyA sequences.

Using the pT2GFP-TKPA as a transfer vector, we constructed the LWS1up2.6kb:GFP and the LWS2up1.8kb:GFP in Figure 2A as the followings. The region from LWS1up2.6kb to GFP in the LWS1/GFP-PAC(E) clone and the region from LWS2up1.8kb to GFP in the LWS2/GFP-PAC(E) clone were isolated by PCR using forward primers harboring a SalI site and reverse primers harboring a NotI site (“LWS1up2.6kb:GFP” and “LWS2up1.8kb:GFP” in Table S2). A DNA segment in the pT2GFP-TKPA from SalI in MCS to NotI in the junction between GFP and HSV-TK polyA was replaced with those segments isolated from the PAC constructs through restriction digestion and ligation at the SalI and NotI sites. In the resulting constructs, the region from LWS1up2.6kb to GFP and that from LWS2up1.8kb to GFP were connected to the HSV-TK polyA (LWS1up2.6kb:GFP and the LWS2up1.8kb:GFP, respectively) at the NotI site.

The LWS1up2.6kb:GFP-LWS2up1.8kb:RFP (Figure 2A) was constructed as follows. The SV40 polyA in the pEGFP-1 vector was isolated together with a NotI site located at its 5′ side by PCR using a forward primer harboring a KpnI site and a reverse primer harboring a SalI site (“SV40-polyA” in Table S2). The isolated fragment was cloned into the pBluescript II (SK-) vector at KpnI and SalI sites. The region from the LWS2up1.8kb to the RFP gene including a NotI site located just downstream of the RFP gene in the LWS1/GFP-LWS2/RFP-PAC(E) clone was isolated with a SalI site attached to the 5′ end of the LWS2up1.8kb. The region was connected to the 3′ side of the SV40 polyA cloned in the pBluescript II (SK-) at SalI site. Then, from the pBluescript construct, the region consisting of the SV40 polyA, LWS2up1.8kb, and RFP gene (from the Not I site at 5′ side of the SV40 polyA to the NotI site at 3′ side of the RFP gene) was inserted into the LWS1up2.6kb:GFP construct in the pT2GFP-TKPA at the NotI site located between the GFP gene and the HSV-TK polyA. This results in the LWS1up2.6kb-GFP segment connected to 5′ side of the SV40 polyA and the LWS2up1.8kb-RFP segment connected to 5′ side of the HSV-TK polyA in the pT2GFP-TKPA (LWS1up2.6kb:GFP-LWS2up1.8kb:RFP).

The LAR:LWS2up1.8kb:GFP and the LAR:*krt8*up564bp:GFP (see Figure 4A and Results section) were constructed as follows. The LAR was isolated from the LWS-PAC(E) clone by PCR using a forward primer harboring a HindIII site and a reverse primer harboring an EcoRI site (“LAR” in Table S2) and was inserted into the HindIII/EcoRI sites in the MCS of pT2GFP-TKPA. For making the LAR:LWS2up1.8kb:GFP, the GFP gene region in the LAR-inserted pT2GFP-TKPA construct (from the SalI site in MCS to the NotI site at the 3′ side of the GFP gene) was replaced with the region from the LWS2up1.8kb to the GFP gene in LWS2up1.8kb:GFP construct in the pT2GFP-TKPA (from the SalI site at the 5′ side of LWS2up1.8kb to the NotI site at the 3′ side of the GFP) by restriction digestion and ligation at the SalI and NotI sites. Similarly, for making the LAR:*krt8*up564bp:GFP, the GFP gene region in the LAR-inserted pT2GFP-TKPA was replaced with the region from the *krt8*up564bp to the GFP gene in LCR:*krt8* construct reported in ref. [41] by restriction digestion and ligation at the SalI and NotI sites.

### Reporter constructs for transient transgenic assay

A series of the GFP-reporter constructs and DNA fragments for the transient transgenic assay (Figure 3B, 3C) were obtained by PCR from LWS-2/GFP-PAC(E) using primers listed in Table S3. These DNA fragments were purified through gel extraction before the microinjection.

### Transgenic fish

Zebrafish were maintained at 28.5°C in a 14-h light/10-h dark cycle as described by ref. [61]. The LWS-PAC derived constructs bearing the I-SceI recognition sequence were injected into the cytoplasm of embryos at the one-cell stage at 20 ng/µl with I-SceI meganuclease (0.5 units/µl) (New England Biolabs, Beverly, MA) in a solution of 0.5× commercial meganuclease buffer with tetramethyl-rhodamin dextran tracer [57].

The reporter constructs in the pT2GFP-TKPA vector were resuspended at a final concentration of 25 ng/µl in 0.1 M KCl and tetramethyl-rhodamin dextran tracer. They were co-injected with mRNA of Tol2 transpsase of 27 ng/µl that was prepared through *in vitro* transcription from pCS-TP using the mMESSAGE mMACHINE kit (Ambion, Austin, TX) [59], [60].

For generation of transgenic lines, the injected embryos were grown to sexual maturity and crossed with non-injected fish in a pair-wise fashion. Founders and fish of subsequent generations transmitting a reporter transgene were screened by PCR-based genotyping as described in ref. [41]. All the transgenic lines analyzed in this study are listed in Table S4.

The GFP-reporter constructs for the transient transgenic assay (Figure 3B) were microinjected with 0.1 M KCl and tetramethyl-rhodamin dextran at a final concentration of 25–50 ng/µl. The LWS2up1.8kb-GFP-*LWS-2* region was injected together with a variety of DNA segments from the LWS1up2.6kb region (Figure 3C) at a final concentration of approximately 25–50 ng/µl each in 0.1 M KCl and tetramethyl-rhodamin dextran tracer.

For transient transgenic assay of GFP expression, embryos injected with the GFP-expression constructs were grown in 0.003% 1-phenyl–2-thiourea after 12–24 hpf to disrupt pigment formation. One eye per injected embryo was examined at 7 dpf for GFP fluorescence under a dissecting fluorescent microscope. The eyes were scored as “+++”, “++”, “+”, and “−” when GFP was expressed in more than 50 cells, in 11–50 cells, in 1–10 cells, and in no cell per eye, respectively [39]–[41].

### Immunohistochemistry

Immunostaining was carried out against adult retinal sections following the procedure of ref. [39]. An antibody against the zebrafish red opsin raised in rabbits [32] was used to stain LDCs. The Cy3-conjugated anti-rabbit IgG was used as a secondary antibody. Images of GFP, RFP and Cy3 fluorescence of the sections were captured using a Zeiss 510 laser-scanning confocal microscope (Zeiss, Thornwood, NY).

## Supporting Information

Figure S1 A transverse section of a Tg(LWS1up2.6kb:GFP-LWS2up1.8kb:RFP)#1464 retina. The dorsal side is oriented at the top of each panel and the ventral side is at the bottom. The left and middle panels show the GFP (green) and RFP (magenta) images, respectively. The right is the merge of the two panels with the DIC image. The sparse expression of RFP in the central to dorsal area is indicated by arrows. Scale bars  = 100 µm.
(2.79 MB TIF)

Figure S2 Expression of GFP in a Tg(LAR:LWS2up1.8kb:GFP)#1501 retina. (A) A transverse section of a Tg(LAR:LWS2up1.8kb:GFP)#1501 retina. The left panel shows the image of GFP signals (green) and the right panel shows the overlay with its DIC image. The dorsal side is oriented at the top of each panel and the ventral side is at the bottom. (B) A vertical and expanded view of the photoreceptor layer of the same retina as shown in (A). GFP (green) was specifically expressed in LDCs, whose outer segments were immunostained with the antibody against the zebrafish red opsin (magenta). Arrowheads indicate the faint GFP signals detected in some bipolar cells. Scale bars  = 100 µm (A), 10 µm (B).
(4.93 MB TIF)

Figure S3 The nucleotide sequence of the LAR. Two OTX (A/GGATTA) and one OTX-like (TGATTA) sequences are underlined with black and gray lines, respectively. The sequence corresponds to the nucleotide position 28416–29048 of GenBank accession number CT573282.6.
(0.71 MB EPS)

Table S1 PCR primers used for modification of the LWS-PAC clones by homologous recombination.
(0.04 MB DOC)

Table S2 PCR primers for DNA constructs used in the Tol2-mediated transgenesis.
(0.03 MB DOC)

Table S3 PCR primers for DNA constructs used in the transient transgenic assay.
(0.04 MB DOC)

Table S4 List of transgenic lines analyzed in this study.
(0.04 MB DOC)
