# Supplementary figures and images for: A Single Enhancer Regulating the Differential Expression of Duplicated Red-Sensitive Opsin Genes in Zebrafish
Source: PLoS Genet. 2010 Dec 16;6(12):e1001245. doi: 10.1371/journal.pgen.1001245 (PMC3002997; doi:10.1371/journal.pgen.1001245)

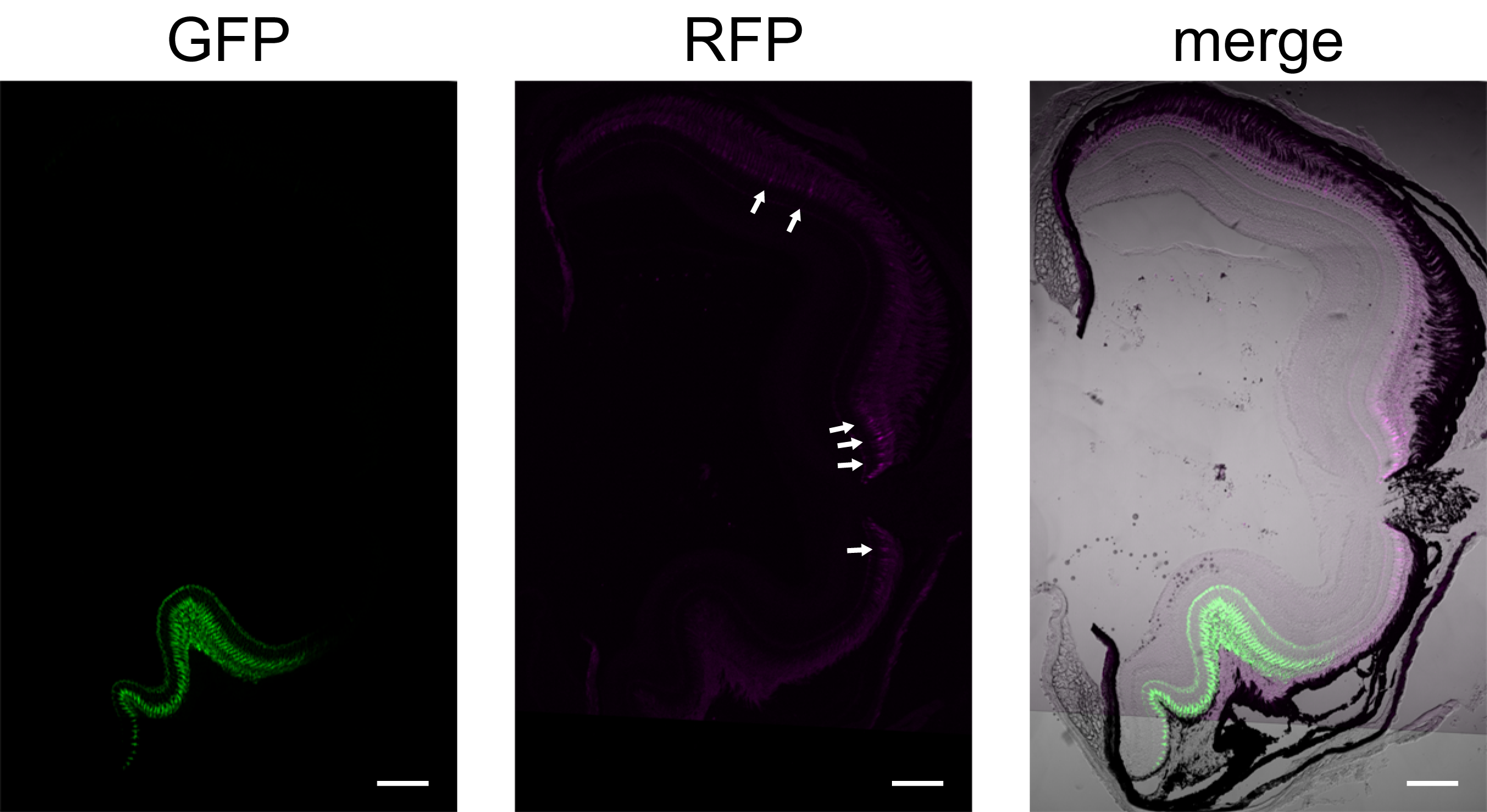

Supplement: Figure S1 — A transverse section of a Tg(LWS1up2.6kb:GFP-LWS2up1.8kb:RFP)#1464 retina. The dorsal side is oriented at the top of each panel and the ventral side is at the bottom. The left and middle panels show the GFP (green) and RFP (magenta) images, respectively. The right is the merge of the two panels with the DIC image. The sparse expression of RFP in the central to dorsal area is indicated by arrows. Scale bars = 100 µm. (2.79 MB TIF) [file pgen.1001245.s001.tif]

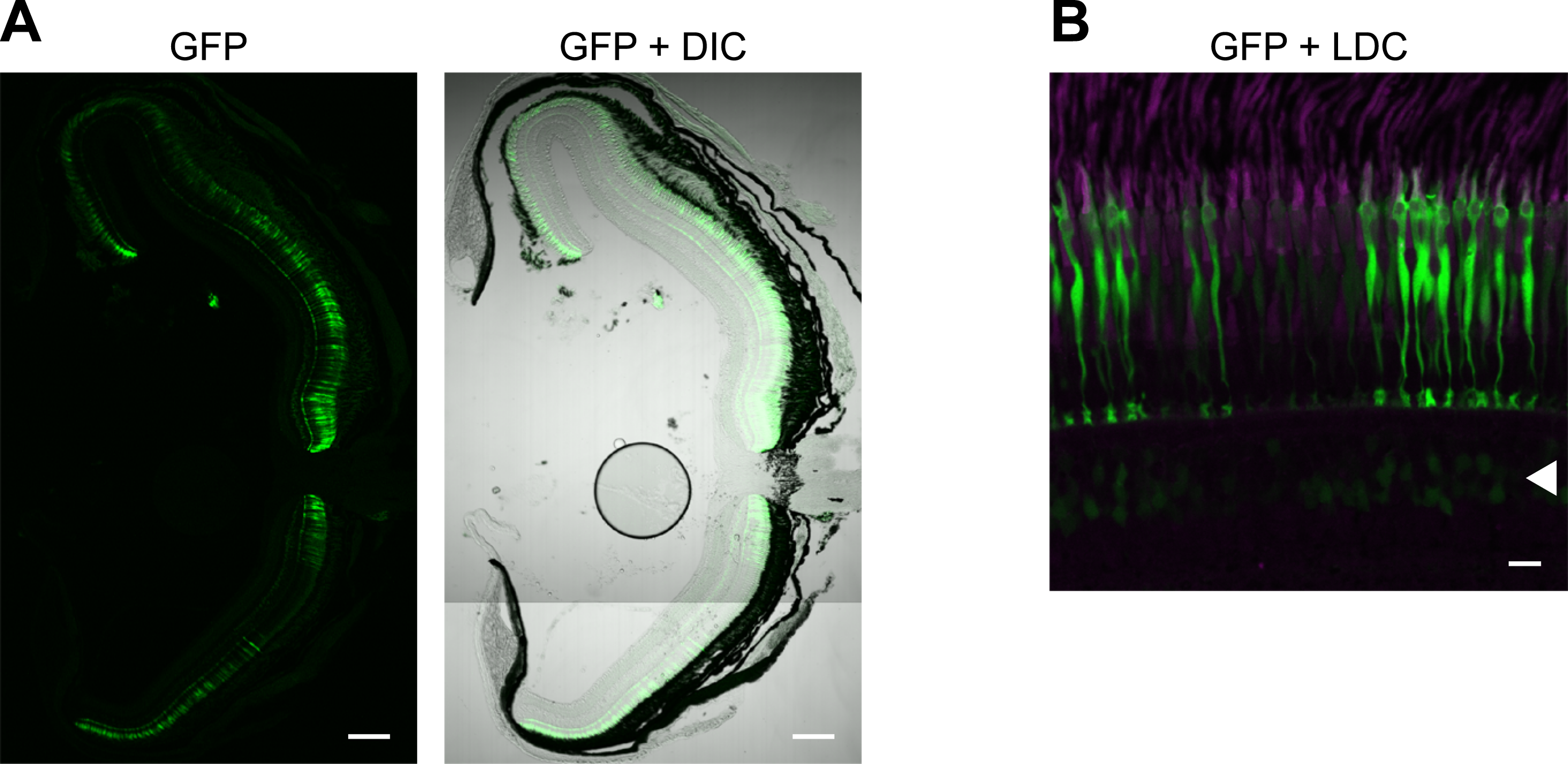

Supplement: Figure S2 — Expression of GFP in a Tg(LAR:LWS2up1.8kb:GFP)#1501 retina. (A) A transverse section of a Tg(LAR:LWS2up1.8kb:GFP)#1501 retina. The left panel shows the image of GFP signals (green) and the right panel shows the overlay with its DIC image. The dorsal side is oriented at the top of each panel and the ventral side is at the bottom. (B) A vertical and expanded view of the photoreceptor layer of the same retina as shown in (A). GFP (green) was specifically expressed in LDCs, whose outer segments were immunostained with the antibody against the zebrafish red opsin (magenta). Arrowheads indicate the faint GFP signals detected in some bipolar cells. Scale bars = 100 µm (A), 10 µm (B). (4.93 MB TIF) [file pgen.1001245.s002.tif]
