# Supplementary material for: A Single Enhancer Regulating the Differential Expression of Duplicated Red-Sensitive Opsin Genes in Zebrafish
Source: PLoS Genet. 2010 Dec 16;6(12):e1001245. doi: 10.1371/journal.pgen.1001245 (PMC3002997; doi:10.1371/journal.pgen.1001245)
Supplement: Table S1 — PCR primers used for modification of the LWS-PAC clones by homologous recombination. (0.04 MB DOC) [file pgen.1001245.s004.doc]

**Table S1.** PCR primers used for modification of the LWS-PAC clones by homologous recombination

| PCR products | primers |
| --- | --- |
| I-SceI-Amp^r^-I-SceI | 5’ TAGGGATAACAGGGTAATGGTCTGACGCTCAGTGGAAC 3’ |
|  | 5’ ATTACCCTGTTATCCCTAAGACGAAAGGGCCTCGTGATAC 3’ |
| Km^r^<>I-SceI-Amp^r^-I-SceI | 5’ GCGCTGAGGTCTGCCTCGTGAAGAAGGTGTTGCTGACTCATACCAGCAGGAATTCGATTAGGGATAAC 3’ |
|  | 5’ AAAGCCACGTTGTGTCTCAAAATCTCTGATGTTACATTGCACAAGTCGATAAGCTTGATATTACCCTG 3’ |
| CAT | 5’ AATGCTTAAGCGAATAAATACCTGTGACGG 3’ |
|  | 5’ GTTTCTTAAGAACTGGCCTCAGGCATTTG 3’ |
| Km^r^ | 5’ CATTCTTAAGTCTCAAAATCTCTGATGTTA 3’ |
|  | 5’ CTACCTTAAGCCTGAATCGCCCCATCATC 3’ |
| LWS-1<>GFP-polyA-CAT | 5’ CAGCTAAGTGACTACAGGTTTGGGCTATACAACAAACCCCAAAAAATGGTGAGCAAGGGCGAGGAG 3’ |
|  | 5’ ATCAAATTACAAATATTTGTGTGAAAAAAGTTGAAAGACTCACCCAACTGGCCTCAGGCATTTGAG 3’ |
| LWS-2<>RFP-polyA-Km^r^ | 5’ AATGGAAATAATTCTTTTATTTATTTGATCTTCAGGGTTTCCAAAATGGTGCGCTCCTCCAAGAAC 3’ |
|  | 5’ ACATGATTTATCCAGCTGTTCCTGAAGATTAAGTTAGTATTTTACCCTGAATCGCCCCATCATCC 3’ |
| LWS-2<>GFP-polyA-CAT | 5’ AATGGAAATAATTCTTTTATTTATTTGATCTTCAGGGTTTCCAAAATGGTGAGCAAGGGCGAGGAG 3’ |
|  | 5’ ATGATTTATCCAGCTGTTCCTGAAGATTAAGTTAGTATTTTACCCAACTGGCCTCAGGCATTTGAG 3’ |
| FRT-CAT-FRT | 5’ GAAGTTCCTATTCTCTAGAAAGTATAGGAACTTCCGAATAAATACCTGTGACGGAAG 3’ |
|  | 5’ GAAGTTCCTATACTTTCTAGAGAATAGGAACTTCAACTGGCCTCAGGCATTTGAGAAG 3’ |
| LAR<>FRT-CAT-FRT | 5’ TTTTTAAAAAGGAATAGAAAAAAAAGTTTACAGACTGCTTTTGTCTTGATGAAGTTCCTATTCTCTAGA 3’ |
|  | 5’ TGTTCCAGTTTAGGGTTTAAAGGTAAATAATTTTAGGATTTAAGAATTCGATGAAGTTCCTATACTTTC 3’ |
