# Supplementary material for: A Single Enhancer Regulating the Differential Expression of Duplicated Red-Sensitive Opsin Genes in Zebrafish
Source: PLoS Genet. 2010 Dec 16;6(12):e1001245. doi: 10.1371/journal.pgen.1001245 (PMC3002997; doi:10.1371/journal.pgen.1001245)
Supplement: Table S2 — PCR primers for DNA constructs used in the Tol2-mediated transgenesis. (0.03 MB DOC) [file pgen.1001245.s005.doc]

**Table S2.** PCR primers for DNA constructs used in the Tol2-mediated transgenesis

| PCR products | Primers |
| --- | --- |
| HSV-TK-polyA | 5' TCGGGCGGCCGCCCACCCTAGGGGGAGGCT 3' |
|  | 5' GTCAGATCTGAGGCTATGGCAGGGCCTG 3' |
| LWS1up2.6kb:GFP | 5' CCAGAGGTCGACACACAGTTCTCATGTAACCTC 3' |
|  | 5' CTGGCGGCCGCTTTACTTGTACAGCTCGTC 3' |
| LWS2up1.8kb:GFP | 5' CTGGGTCGACGTTGTGCACCAGATCTGAGTC 3' |
|  | 5' CTGGCGGCCGCTTTACTTGTACAGCTCGTC 3' |
| SV40-polyA | 5’ GGTACCGCGGCCGCGACTCTAGATCA 3’ |
|  | 5’ CCAAGTCGACATACATTGATGAGTTTGGACAAACC 3’ |
| LAR | 5' CCCCAAGCTTTTGGTCTTGGCTCCATCCCC 3' |
|  | 5' CGTGAATTCTCTCTGTATAATGGCACTTGC 3' |
