# Supplementary material for: A Single Enhancer Regulating the Differential Expression of Duplicated Red-Sensitive Opsin Genes in Zebrafish
Source: PLoS Genet. 2010 Dec 16;6(12):e1001245. doi: 10.1371/journal.pgen.1001245 (PMC3002997; doi:10.1371/journal.pgen.1001245)
Supplement: Table S3 — PCR primers for DNA constructs used in the transient transgenic assay. (0.04 MB DOC) [file pgen.1001245.s006.doc]

**Table S3.** PCR primers for DNA constructs used in the transient transgenic assay

| PCR products | Primers |
| --- | --- |
| SWS2up5.2kb | 5’ CTGCAGTAATCTTGGTGATGGGAAATTTAA 3’ |
|  | 5’ GTTCAACAGGAGCTATAAATCACGTAAGAT 3’ |
| LWS1up2.6kb | 5’ ACACAGTTCTCATGTAACCTCATCCTGCGGACGAA 3’ |
|  | 5’ GTTCAACAGGAGCTATAAATCACGTAAGAT 3’ |
| LWS1up2.6kb(pA) | 5’ ACACAGTTCTCATGTAACCTCATCCTGCGGACGAA 3’ |
|  | 5’ TGATGAGTTTGGACAAACCACAACTAGAAT 3’ |
| LWS1up1.9kb | 5’ GAGAGAGAATGTGATGTTAGCTTTTGCCAT 3’ |
|  | 5’ GTTCAACAGGAGCTATAAATCACGTAAGAT 3’ |
| LWS1up1.3kb | 5’ TTGGTCTTGGCTCCATCCCCGTCCTGGTTT 3’ |
|  | 5’ GTTCAACAGGAGCTATAAATCACGTAAGAT 3’ |
| LWS1up0.6kb | 5’ AGCAAGTGCCATTATACAGAGAGTCTTAAA 3’ |
|  | 5’ GTTCAACAGGAGCTATAAATCACGTAAGAT 3’ |
| LWS1(ATG) | 5’ AGGTTTGGGCTATACAACAAACCCCAAAAA 3’ |
|  | 5’ GTTCAACAGGAGCTATAAATCACGTAAGAT 3’ |
| LWS2up1.8kb | 5’ GTTGTGCACCAGATCTGAGTCAGACATGGGGAAAA 3’ |
|  | 5’ GTTCAACAGGAGCTATAAATCACGTAAGAT 3’ |
| LWS1up2.6-0kb | 5’ ACACAGTTCTCATGTAACCTCATCCTGCGGACGAA 3’ |
|  | 5’ ATGCTCGGATCCTTTTTGGGGTTTGTTGTATAG 3’ |
| LWS1up2.6-0.6kb | 5’ ACACAGTTCTCATGTAACCTCATCCTGCGGACGAA 3’ |
|  | 5’ ATGGCAGGATCCGCTTACTGTAGGAGTAAATAA 3’ |
| LWS1up2.6-1.3kb | 5’ ACACAGTTCTCATGTAACCTCATCCTGCGGACGAA 3’ |
|  | 5’ AGCCAAGGATCCGACAAAAGCAGTCTGTAAACTT 3’ |
| LWS1up1.9-0.6kb | 5’ GAGAGAGAATGTGATGTTAGCTTTTGCCAT 3’ |
|  | 5’ ATGGCAGGATCCGCTTACTGTAGGAGTAAATAA 3’ |
| LWS1up1.9-1.3kb | 5’ GAGAGAGAATGTGATGTTAGCTTTTGCCAT 3’ |
|  | 5’ AGCCAAGGATCCGACAAAAGCAGTCTGTAAACTT 3’ |
| LWS1up1.3-0.6kb | 5’ TTGGTCTTGGCTCCATCCCCGTCCTGGTTT 3’ |
|  | 5’ ATGGCAGGATCCGCTTACTGTAGGAGTAAATAA 3’ |
