# Supplementary material for: A Single Enhancer Regulating the Differential Expression of Duplicated Red-Sensitive Opsin Genes in Zebrafish
Source: PLoS Genet. 2010 Dec 16;6(12):e1001245. doi: 10.1371/journal.pgen.1001245 (PMC3002997; doi:10.1371/journal.pgen.1001245)
Supplement: Table S4 — List of transgenic lines analyzed in this study. (0.04 MB DOC) [file pgen.1001245.s007.doc]

**Table S4**. List of transgenic lines analyzed in this study.

| Name | Method | Line ID | Reporter expression | Figure |
| --- | --- | --- | --- | --- |
| Tg(LWS1/GFP-LWS2/RFP-PAC(E)) | I-SceI | #1229 | + | 1B |
| Tg(LWS1/GFP-LWS2/RFP-PAC(H)) | I-SceI | #430 | + | 1C, 5B |
| Tg(LWS1up2.6kb:GFP-LWS2up1.8kb:RFP) | Tol2 | #1464 | + | S1 |
|  |  | #1631 | + | 2B, C |
|  |  | #1640 | + |  |
| Tg(LWS1up2.6kb:GFP) | Tol2 | #1508 | + |  |
|  |  | #1509 | + | 2D-F |
|  |  | #1515 | + |  |
| Tg(LWS2up1.8kb:GFP) | Tol2 | #1433 | - |  |
| Tg(LAR:LWS2up1.8kb:GFP) | Tol2 | #1481 | - |  |
|  |  | #1491 | - |  |
|  |  | #1496 | - |  |
|  |  | #1499 | + | 4B-D |
|  |  | #1501 | + | S2 |
| Tg(LAR:*krt8*up564bp:GFP) | Tol2 | #1469 | - |  |
|  |  | #1477 | - |  |
| Tg(ΔLAR-LWS1/GFP-LWS2/RFP-PAC(E)) | I-SceI | #1143 | + | 5C |
|  |  | #1166 | + |  |
| Tg(ΔLAR-LWS1/GFP-LWS2/RFP-PAC(H)) | I-SceI | #1107 | + | 5D, E |
|  |  | #1100 | + |  |
